# Empirical evaluation of prediction intervals for cancer incidence

**DOI:** 10.1186/1471-2288-5-21

**Published:** 2005-06-10

**Authors:** Bjørn Møller, Harald Weedon-Fekjær, Tor Haldorsen

**Affiliations:** 1 Cancer Registry of Norway, Montebello, N-0310 Oslo, Norway

## Abstract

**Background:**

Prediction intervals can be calculated for predicting cancer incidence on the basis of a statistical model. These intervals include the uncertainty of the parameter estimates and variations in future rates but do not include the uncertainty of assumptions, such as continuation of current trends. In this study we evaluated whether prediction intervals are useful in practice.

**Methods:**

Rates for the period 1993–97 were predicted from cancer incidence rates in the five Nordic countries for the period 1958–87. In a Poisson regression model, 95% prediction intervals were constructed for 200 combinations of 20 cancer types for males and females in the five countries. The coverage level was calculated as the proportion of the prediction intervals that covered the observed number of cases in 1993–97.

**Results:**

Overall, 52% (104/200) of the prediction intervals covered the observed numbers. When the prediction intervals were divided into quartiles according to the number of cases in the last observed period, the coverage level was inversely proportional to the frequency (84%, 52%, 46% and 26%). The coverage level varied widely among the five countries, but the difference declined after adjustment for the number of cases in each country.

**Conclusion:**

The coverage level of prediction intervals strongly depended on the number of cases on which the predictions were based. As the sample size increased, uncertainty about the adequacy of the model dominated, and the coverage level fell far below 95%. Prediction intervals for cancer incidence must therefore be interpreted with caution.

## Background

Prediction of cancer incidence is of great interest for both health authorities and the scientific community [1]. Estimates of the future cancer burden should indicate the appropriate amounts of resources that will be needed for diagnosis, treatment and rehabilitation. Since quantification of future cancer incidence is inherently uncertain [2], some measurement of the uncertainty would be useful. It has been suggested that, similar to the confidence intervals calculated in standard statistical modeling, prediction intervals should be presented with predictions of cancer incidence [3,4].

When future cancer incidence is predicted in a statistical model, three sources of uncertainty are associated with the predicted numbers. The first is the variance of the parameters estimated in the model; the second is the random variation of the future number of cases; and the third is the adequacy of the model. The last includes the uncertainty in both the mathematical structure of the model and the choice of projected components, such as whether to assume that current trends will continue into the future. The first two sources of variance can be included in a statistical model. Intervals that are based only on the variance of the parameters estimated in the model are called 'confidence intervals', while intervals that also encompass variation in the future number of cases are called 'prediction intervals'.

The third source of uncertainty, variations caused by deviations from the model assumptions, is difficult to formalize. Engeland et al. [5] argued that "The large uncertainties associated with the specification of the models used in the predictions obviate the construction of confidence intervals." One way of investigating the extent of the uncertainty in specification of a model is to calculate prediction intervals from historical data and then calculate the proportion of those intervals that actually cover the observed number of cancer cases. If the proportion is close to the nominal level, e.g. 95 %, the prediction intervals could be taken to give a fair description of the range of likely values to be expected. A low proportion would indicate that there was great uncertainty in the model assumptions, and that prediction intervals should be interpreted with caution. The aim of this study was to evaluate the extent to which prediction intervals derived from incidence rates can be expected to cover the actual numbers observed 10 years later.

## Methods

### Material

The material consisted of new cases of cancer reported to the cancer registries of Denmark, Finland, Iceland, Norway and Sweden between 1958 and 1997, and population figures covering the same calendar period from the central statistical offices in these countries. Sweden has the largest population, with 8.9 million inhabitants, followed by Denmark, Finland, Norway and Iceland with 5.3, 5.2, 4.5 and 0.3 million, respectively. The Nordic cancer registries receive reports from physicians, hospitals, pathological and cytological laboratories and (except in Sweden) death certificates [6]. Compulsory reporting and information from multiple sources ensure almost 100% completeness of all the cancer registries [7].

Table 1 lists the cancer sites included in the study. Detailed descriptions of the types of tumors at each site that are included was given by Engeland et al. [5]. As there were few cases of cancers of the lip and larynx among women and of the breast among men, these cancers were included in 'other sites'.

**Table 1:** Proportions of observed numbers of cases in 1993–97 covered by prediction intervals based on trends up to 1987, by site, sex, country and frequency*.

	Number of intervals	Coverage number (%)	P-value for difference
Site			0.54
Lip	5	2 (40)	
Tongue, oral cavity and pharynx	10	5 (50)	
Oesophagus	10	5 (50)	
Stomach	10	5 (50)	
Colon	10	7 (70)	
Rectum	10	5 (50)	
Pancreas	10	6 (60)	
Larynx	5	3 (60)	
Lung	10	4 (40)	
Breast	5	2 (40)	
Cervix uteri	5	2 (40)	
Corpus uteri	5	2 (40)	
Ovary	5	1 (20)	
Prostate	5	2 (40)	
Testis	5	4 (80)	
Kidney	10	7 (70)	
Urinary bladder	10	5 (50)	
Melanoma of the skin	10	3 (30)	
Thyroid	10	7 (70)	
Non-Hodgkin lymphomas	10	4 (40)	
Hodgkin disease	10	9 (90)	
Multiple myeloma	10	4 (40)	
Acute leukaemia	10	7 (70)	
Other sites	10	3 (30)	
Sex			0.67
Male	100	50 (50)	
Female	100	54 (54)	
Country			<0.001
Denmark	40	18 (45)	
Finland	40	21 (53)	
Iceland	40	35 (88)	
Norway	40	20 (50)	
Sweden	40	10 (25)	
Frequency			<0.001
≤ 70 per year	50	42 (84)	
> 70 and ≤ 230 per year	50	26 (52)	
> 230 and ≤ 555 per year	50	23 (46)	
> 555 per year	50	13 (26)	

*Average number of cases per year in the period 1983–87, grouped into four quartiles.

The data were tabulated in five-year age groups (0–4, 5–9, ..., 80–84, ≥85) and five-year calendar periods (1958–62, 1963–67, ..., 1993–97). Since there were 20 types of cancers for each sex and five countries in the study, we had 200 different combinations for which to make predictions.

### Statistical model

The age-period-cohort (APC) model [8] has been widely used for predicting cancer incidence and mortality [9-13]. This Poisson regression model is based on tables with five-year age groups and five-year calendar periods. Birth cohorts were constructed synthetically by subtracting age from period. The model can be written as

*R*_*ap *_= exp(*A*_*a *_+ *D*·*p *+ *P*_*p *_+ *C*_*c*_),

where *R*_*ap *_is the incidence rate in age group *a *in calendar period *p*, *D *is the common drift parameter [14], *A*_*a *_is the age component for age group *a*, *P*_*p *_is the non-linear period component of period *p *and *C*_*c *_is the non-linear cohort component of cohort *c*, *c *= *p *- *a*. We used a slightly modified model [5], substituting a power link for the log link:

*R*_*ap *_= (*A*_*a *_+ *D*·*p *+ *P*_*p *_+ *C*_*c*_)^5^,

in order to level off the exponential growth in the multiplicative model. An empirical study of these two models showed that the power model gave predictions that were closer to the observed rates [2]. For some cancer sites for which there were only a few cases in Iceland, a model without the cohort component was used; see Engeland et al. [5] for details and on the lower limits of the age groups included for each site in all the countries.

To ensure a reasonable fit of each data set to the model, the number of five-year periods on which the predictions should be based on was chosen. First, a model including the last six 5-year periods (1958–1987) was fitted. If the model was rejected by a test for goodness-of-fit (5% level), a model including the last five periods was fitted. If this model was also rejected, only the last four periods were used.

Future non-linear effects of cohort and period were assumed to be equal to the last estimated effect in the model, and predictions were made by projecting the drift. Numbers of cancer cases were predicted by multiplying the predicted rates by the person-years at risk in a given age group and time period.

### Prediction intervals, coverage level and discrepancy ratio

In an article on the precision of cancer incidence predictions, Hakulinen and Dyba [3] derived prediction intervals for Poisson distributed variables. Following their paper, let , where *a *is the age group, *f *is the future calendar period for which predictions are to be made (*f *= 8 which corresponds to the period 1993–97), *R*_*af *_is the incidence rate in age group *a *in period *f*, and *k*_*af *_and *n*_*af *_are the corresponding number of cases and person-years, respectively. Further, let the expected number of cases, E(*k*_*af*_), be defined as *λ*_*af *_= *n*_*af *_(*A*_*a *_+ *D*·*P *+ *P*_*f *_+ *C*_*c*_)^5^, and let  and . The variance of the future number of cases, var (*k*_*f*_), can be written as var (*k*_*f*_) = var () + *σ*^2 ^E(). The first component, var (), reflects the uncertainty in estimating the parameters in the model. The second component, *σ*^2 ^E(), reflects the random variation of the future number of cases for a Poisson-distributed variable, allowing for extra-Poisson variation, i.e., *σ*^2 ^measures the degree of over-dispersion.

An estimator for the variance of the future number of cases can be found by using Taylor series expansion of non-linear functions (see formulas in the appendix). A 95% prediction interval for the future number of cancer cases, *k*_*f*_, can then be calculated from an assumption of normality:



where *k*_*f *_was estimated by .

On the basis of up to six 5-year periods between 1958 and 1987, prediction intervals for the numbers of cases in the period 1993–97 were calculated for all 200 combinations of 20 sites for each sex in each of the five countries. The coverage level was defined as the proportion of the prediction intervals that covered the observed number of cases in 1993–97.

**Figure 1:**
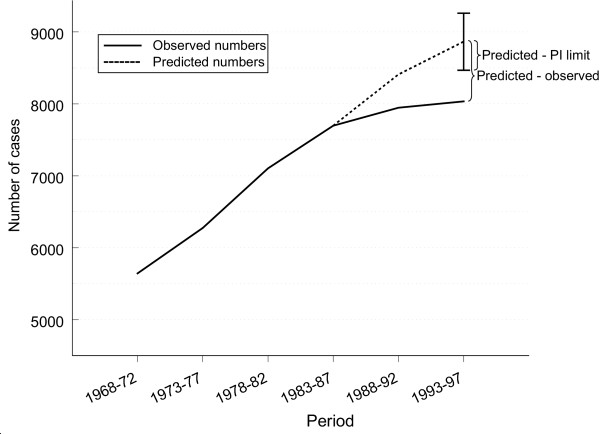
**The discrepancy ratio**. Illustration of the components of the discrepancy ratio. The discrepancy ratio compares the distance between predicted and observed number of cases with the distance between predicted number and the limit of the prediction interval.

The coverage level only indicates whether the observed number of cases was inside the prediction interval or not. Additional information can be gained by looking at how far outside of the interval the observations fall. Discrepancy ratio was defined as the absolute distance between observed and predicted number of cases, measured in half prediction interval widths:



where  and *k*_*f *_are the predicted and observed number of cases, respectively, and  is the distance between predicted number of cases and the limit of the prediction interval. Figure 1 illustrates the discrepancy ratio. When the discrepancy ratio is larger than 1, it measures how much wider the prediction interval had to be to cover the observed number of cases. In Figure 1, the observed number of cases is about twice as far from the predicted number compared to the lower limit of the prediction interval, giving a discrepancy ratio of 2.

Fisher's exact test was used to evaluate differences between sites, countries and quartiles of number of cases. A binomial regression model was used to study the effect of country and frequency simultaneously.

## Results

Prediction intervals were calculated from data up to 1987 for the 200 combinations of sex, site and country. After observing the number of cases 10 years later, in the period 1993–97, 104 (52%) of the observed numbers were covered by the prediction intervals. Coverage levels for specific sites varied from 20% to 90%, but only five or ten intervals were calculated for each site and the difference between the sites was not statistically significant (Table 1).

The coverage levels for the five Nordic countries varied widely. For the country with the smallest population, Iceland, the coverage level was 88%, which is relatively close to the nominal level of 95%. The levels for Denmark, Finland and Norway were around 50 %, while that for Sweden, the most populous country, was only 25%. When the 200 different predictions were ranked according to the annual number of cases in the period 1983–87 (frequency), the cut-offs for the four quartiles were 70, 230 and 555. Subdividing the predictions according to these quartiles, the coverage level decreased markedly with the annual number of cases, being 84% for the first quartile and 52%, 46% and 26% for the next three, respectively (Table 1).

Table 2 shows the associations between country and coverage level as odds ratios (ORs), where the odds of covering the observed number of cases with the prediction intervals in each country was calculated relative to the odds in Denmark. The crude numbers reflect the pattern seen in Table 1, the chance of the observed number of cases being within the prediction interval being similar in Denmark, Finland and Norway, higher in Iceland and lower in Sweden. In the binomial regression model the logarithm of the annual number of cases in 1983–87 was used instead of the frequency itself or the quartiles of the numbers. The reason for this was that the fit to the model was worse when the frequency variable was entered on a linear rather than on a log-linear scale. Also, entering the variable as four categories instead of as a continuous variable did not further improve the fit. The OR for the logarithm of the frequency was 0.52, indicating that an increase of one unit on the log scale of the frequency reduced the chance of covering the observed number of cases by about 50%. When country and frequency were mutually adjusted for in a multiple binomial regression analysis, the effect of country declined and became non-significant, while the effect of frequency remained almost unchanged. In the univariate analysis, the OR for Iceland relative to Denmark was 8.5 (95 % confidence interval (CI): 2.8 – 26), which was reduced to 1.7 (95 % CI: 0.4 – 7.6) in the adjusted analysis. The coverage level for Sweden remained lower, with OR = 0.49 (95 % CI: 0.2 – 1.3) for Sweden relative to Denmark.

**Table 2:** Odds ratios (ORs) with corresponding 95% confidence intervals (CIs) for covering the observed number of cases in the period 1993–97 with the prediction intervals calculated from trends up to 1987.

	Crude			Adjusted*		
	OR	95 % CI	P-value	OR	95 % CI	P-value
Country			< 0.001			0.39
Denmark	1.00	Reference		1.00	Reference	
Finland	1.35	0.56 – 3.2		1.18	0.47 – 2.9	
Iceland	8.54	2.8 – 26.3		1.72	0.39 – 7.6	
Norway	1.22	0.51 – 2.9		1.08	0.43 – 2.7	
Sweden	0.41	0.16 – 1.1		0.49	0.18 – 1.3	
Log(frequency)^§^	0.52	0.41 – 0.66	< 0.001	0.60	0.44 – 0.83	0.02

*Both variables included in the model.

^§^Logarithm of average number of cases per year in the period 1983–87.

**Figure 2:**
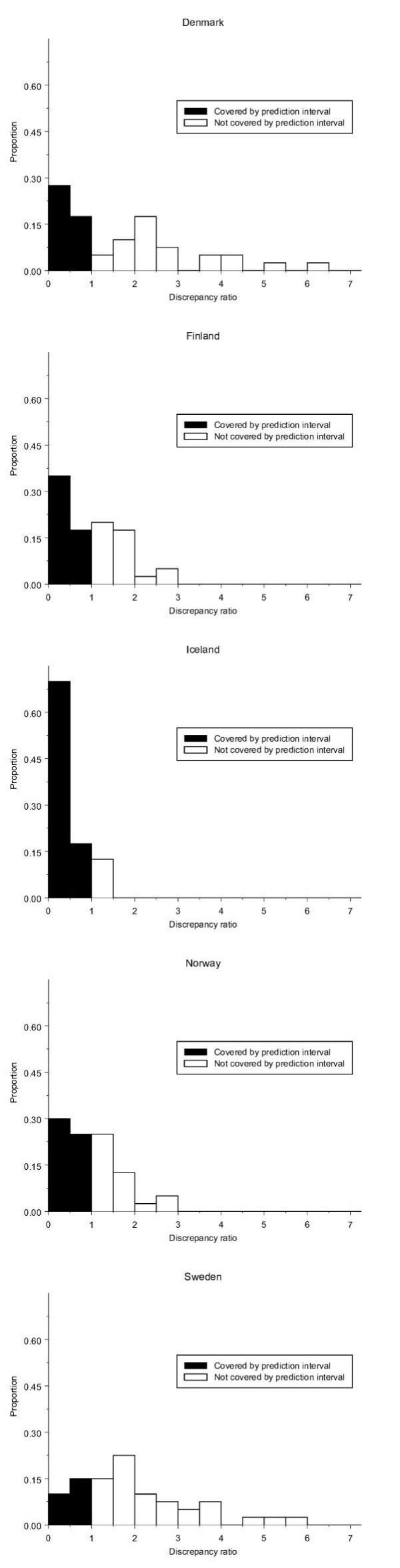
**Empirical distribution of discrepancy ratio**. Empirical distribution of discrepancy ratio by country. Predictions for the 40 combinations of 20 sites for each sex constitute the distribution in each country.

The distribution of the discrepancy ratio for each country is plotted in Figure 2. For Iceland, most of the discrepancy ratios were below 1, corresponding to a coverage level of 88%. Of the 12 % with a higher discrepancy ratio, none was more than 50 % outside of the prediction interval. In Finland and Norway, observed numbers of cases fell up to tree times further from the predicted numbers compared to the limits of the prediction intervals, while in Denmark and Sweden some predictions were 5–6 times outside of the intervals.

## Discussion

**Figure 3:**
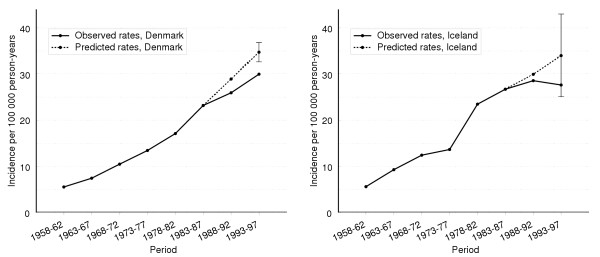
**Illustration of prediction interval**. Age standardized (World population) incidence rates of lung cancer among women in Iceland and Denmark. Predicted rates based on observed rates up to 1987, with corresponding 95% prediction interval for the period 1993–97 for each country.

The coverage levels in the five Nordic countries differed mainly as a function of the numbers of cases that formed the basis for the predictions. In Iceland, where there were generally few cases, the coverage level calculated from 95% prediction intervals was 88% while that in the other Nordic countries, which had much more cases, was of only 25–53%. The problem associated with interpretation of prediction intervals is illustrated in Figure 3, where the observed age-standardized (World standard [15]) incidence rates for cancer of the lung in women in Iceland and Denmark are plotted against the predicted rates, with 95 % prediction intervals. Although the difference between the observed and predicted rates was smaller in Denmark than in Iceland, the wide prediction interval for Iceland meant that the observed rate in 1993–97 was covered by the interval constructed for Iceland, but the narrower interval for Denmark failed to cover the observed rate for that country.

A statistical model is only a simplification of true underlying associations between variables. The finding that the coverage level is inversely proportional to the sample size can be explained by considering the difference between the modeled (simplified) and true (complex) relationship between the cancer rate and the explanatory variables age, period and cohort. When the sample size increases, deviations between the modeled and true relationship will dominate, and non-overage will become a problem. It is useful to distinguish between calculations of prediction intervals for values within the observed range of values of the explanatory variables (interpolation), and outside the range (extrapolation). The non-coverage problem is even larger for extrapolations, because they also relay on the assumption of continuation of current trends. Another difference between interpolation and extrapolation is that when the sample size increases, the possibility to improve the model increases. This would then reduce the non-coverage problem for interpolations, by reducing the distance between the true and the modeled relationship between the variables. Extrapolations, on the other hand, consist of making predictions for values of the covariates outside the range of observed values. Thus, we have to make assumptions that cannot be evaluated from the observed data and the problem with non-coverage for extrapolations are not necessarily reduced by improvements in the model.

It could be argued that a low coverage level indicates that the model is not appropriate, rather than that calculation of predication intervals is per se misleading. With the possible exception of tobacco and lung cancer, few associations are strong enough to be modelled directly. Instead, we used calendar period and birth cohort as proxy variables for changes in underlying risk factors in the model. Møller et al. [2] found that the method used in this study performed fairly well in comparison with other methods currently in use for predicting cancer incidence, and that all the methods evaluated missed the observed number of cases by 10–15% on average for 10 year predictions. Prediction methods could therefore be improved to increase the overall coverage level, although it would be unreasonable to expect that the correct model, or close to it, could be specified for all cancer sites. As long as predictions are based on some type of extrapolation from a statistical model, the coverage level will generally decrease as a function of sample size. The problem is that at the time when the prediction intervals are calculated, the appropriateness of the model with respect to extrapolation into the future usually cannot be evaluated. Prediction intervals can thus be misleading, if they are interpreted as the range of likely values for the number of cases to be expected.

The number of cases from which the predictions for the different cancer sites were made varied widely. Cancers at some sites are very common, like those of the prostate, lung and breast, while others occur less frequently. Because the coverage levels vary with frequency, we would also have expected them to vary by site. The differences were not, however, statistically significant, probably because of the small number of prediction intervals calculated for each site.

A relatively large sample size was associated with a narrow prediction interval, as seen for lung cancer among women in Denmark (Figure 3). The prediction intervals are based on asymptotic theory, which can result in underestimates of variance. Bootstrapping is a suitable method for investigating this problem [16]. We constructed a 95% prediction interval by bootstrapping the data for lung cancer among women in Denmark, assuming that each cell followed a binomial distribution in which the incidence rate and the number of person-years at risk were used as probability of success and number of trials, respectively. We re-sampled the data 1000 times, calculating the predicted world-standardized incidence rate each time. The 95% bootstrap interval, calculated by selecting the 2.5 and 97.5 percentiles of the 1000 predictions, was 32.7 – 36.7, which is fairly close to the asymptotic interval of 32.6–36.8. This indicates that the asymptotic intervals calculated in this study describe the uncertainty in the predicted number of cases well, given a correctly specified model.

Population forecasts are needed for predicting the number of cancer cases. In this paper, we assumed that these numbers were known, but in reality they constitute a separate source of uncertainty. Population forecasts are themselves extrapolations, relying on assumptions about future migration patterns, birth rates and death rates. If our projections for 1993–97 had been calculated from population forecasts in 1987, the coverage levels would probably have been even lower.

Most of the differences in coverage level among the five countries disappeared when the number of cases was controlled for. The remaining difference was not significant, but the probability of covering the rates with prediction intervals for Sweden continued to be lower after adjustment of sample size. Møller et al. [2] showed that the predictions for Sweden were more different from the observed number of cases in 1993–97 than those in the other countries, measured as the median of the absolute value of the relative difference between the predicted and observed numbers of cases. This explains the lower coverage level for Sweden, and indicates that the trends current in 1987 continued to a lesser extent in Sweden than in the other countries.

Engeland and co-workers [5] were reluctant to include prediction intervals with their predictions of cancer incidence in the Nordic countries. A similar view was expressed both with regard to an update of predictions for the Nordic countries [17], and to a prediction of cancer incidence in New South Wales, Australia [18]. There are, however, some instances where prediction intervals can be of value. In cancer surveillance, inclusion of prediction intervals in a routine comparison of the latest observed rates with rates predicted from previous trends, can help to identify changes in the rates beyond random variation. The potential reasons for any discrepancy between the observed and predicted rates can then be studied, including changes in risk factors, diagnostic methods or interventions such as screening programs. In Finland, predicted values with prediction intervals for 1980 were calculated based on rates up to 1968 and compared to observed number of cases in 1980 [19]. Of 33 prediction intervals, 22 (67%) covered the observed values, and the authors discussed possible reasons for those cancers where the prediction intervals failed to cover the observed number of cases. Prediction intervals can also be used to identify highly uncertain predictions. For instance, 26 male cancer cases of the lip were predicted in Iceland in the period 1993–97, and the 95 % prediction interval was 6–46 cases.

## Conclusion

We do not recommend use of prediction intervals when the predictions are used for administrative purposes, like planning appropriate amounts of resources for diagnosis, treatment and rehabilitation, as the intervals can give a false impression of the precision of the predictions. When predictions are based on larger numbers of cases, the uncertainty in estimating the parameters of the model and the random variation of the future rates are decreased. Even relatively minor deviations in the assumptions can then result in observed rates in the future that are outside the range of likely values indicated by the prediction interval.

## Appendix

A 95 % prediction interval for the future number of cancer cases, *k*_*f*_, can be calculated on the basis of an assumption of normality:  were *k*_*f *_can be estimated by . The variance of *k*_*f *_can be written , and  and  can be estimated by Taylor series expansion of non-linear functions:



Further, the parameter for over-dispersion, *σ*^2^, can be estimated from the ratio between the residual deviance of the model and the corresponding number of degrees of freedom [20], and  can be estimated by . We used the statistical package R in the calculations, and the variances and covariances of the estimates of the parameters in the model were found in the covariance matrix of the glm-object.



## Competing interests

The author(s) declare that they have no competing interests.

## Authors' contributions

BM designed the study based on discussions with HWF and TH. BM and HWF collected and analyzed the data. BM drafted the initial manuscript. All authors contributed and approved the final manuscript.

## Pre-publication history

The pre-publication history for this paper can be accessed here:


